# The Yeast Ubr1 Ubiquitin Ligase Participates in a Prominent Pathway That Targets Cytosolic Thermosensitive Mutants for Degradation

**DOI:** 10.1534/g3.111.001933

**Published:** 2012-05-01

**Authors:** Farzin Khosrow-Khavar, Nancy N. Fang, Alex H. M. Ng, Jason M. Winget, Sophie A. Comyn, Thibault Mayor

**Affiliations:** Department of Biochemistry and Molecular Biology, Center for High-Throughput Biology, University of British Columbia, Vancouver, BC V6T 1Z4, Canada

**Keywords:** protein quality control, misfolding, ubiquitin proteasome system, E3 ligase, Ubr1, San1, *Saccharomyces cerevisiae*

## Abstract

Mutations causing protein misfolding and proteolysis are associated with many genetic diseases. The degradation of these aberrant proteins typically is mediated by protein-quality control pathways that recognize misfolded domains. Several E3 ubiquitin ligases have been shown to target cytosolic misfolded proteins to the proteasome. In this study, we characterized a panel of more than 20 cytosolic thermosensitive mutants from six essential genes in *Saccharomyces cerevisiae*. These wild-type proteins are stable at restrictive temperature. In contrast, we found that a large portion of the mutants is degraded at nonpermissive temperature in a proteasome-dependent manner. Approximately one-third of the assessed unstable mutants are targeted by the Ubr1 ubiquitin ligase. In two cases, efficient degradation of the thermosensitive mutants is abrogated in the absence of Ubr1 alone, whereas in a third case it is reliant on the dual deletion of Ubr1 and the nuclear E3 ligase San1. We found that the impairment of the degradation of these quality control substrates at the restrictive temperature is associated with the suppression of thermosensitive phenotype. This study confirms that Ubr1 plays an important role in the degradation of cytosolic misfolded proteins and indicates that degradation mediated by protein quality control is a major cause for the conditional lethality of mutated essential genes.

The eukaryotic cell has developed critical degradative protein quality control (QC) pathways to eliminate misfolded and/or damaged polypeptides (Buchberger *et al.* 2010). Failure to remove these aberrant proteins can lead to the formation of oligomers or aggregates that are associated with human proteopathies such as Parkinson and Huntington disease (Hol and Scheper 2008; Roth *et al.* 2008). On the other hand, degradation of misfolded proteins as the result of point mutations can also be detrimental and lead to loss of function diseases, such as cystic fibrosis and phenylketonuria (Cheng *et al.* 1990; Lichter-Konecki *et al.* 1988). In this case, the degradative QC depletes from the cell proteins that are partially misfolded (as a result of mutations) but otherwise functional.

The ubiquitin proteasome system plays a major role in degradative QC, in which misfolded proteins are first conjugated to poly-ubiquitin chains and then recognized and degraded by the proteasome (Finley 2009). Protein ubiquitylation is an ATP-driven process catalyzed by a cascade of three enzymes: E1 (ubiquitin-activating enzyme), E2 (ubiquitin-conjugating enzyme), and E3 (ubiquitin ligases). The conjugation of ubiquitin normally occurs on substrate lysine residues and substrate recognition is typically facilitated by E3 ligases (Pickart and Eddins 2004).

Distinct QC pathways have been identified in different cellular compartments. Perhaps the best characterized of the degradative QC pathways is the endoplasmic reticulum−associated degradation, in which newly synthesized misfolded proteins are recognized by chaperone proteins in the ER, retrotranslocated to the cytoplasm, poly-ubiquitylated, and degraded by the proteasome (Anelli and Sitia 2008; Hirsch *et al.* 2009). In the yeast nucleus, San1 ligase is involved in the poly-ubiquitylation and degradation of nuclear misfolded proteins (Gardner *et al.* 2005; Matsuo *et al.* 2011). In this case, San1 can directly bind to misfolded substrates through its disordered N-terminal and C-terminal domains (Rosenbaum *et al.* 2011).

A majority of proteins are located in the cytoplasm (Huh *et al.* 2003), and several E3 ligases have been shown to target cytosolic misfolded proteins. In mammalian cells, CHIP (carboxyl terminal of Hsc70 interacting protein) is a U-box domain ubiquitin ligase implicated in cytosolic QC. CHIP interacts with Hsp70 and Hsp90 chaperone proteins and ubiquitylates misfolded proteins by targeting them for proteasome degradation (Connell *et al.* 2001; Meacham *et al.* 2001; Qian *et al.* 2006). In *Saccharomyces cerevisiae*, in which CHIP is not conserved, several pathways have been implicated in cytosolic protein QC. For instance, Ubr1 ubiquitin ligase was shown to target misfolded proteins in the cytoplasm (Eisele and Wolf 2008; Heck *et al.* 2010; Nillegoda *et al.* 2010; Prasad *et al.* 2010), a function also conserved in mammalian cells (Sultana *et al.* 2012). The N-end rule Ubr1 ligase has been extensively characterized for its ability to recognize and target for degradation polypeptides with destabilizing N-terminal amino acids, such as arginine (Bartel *et al.* 1990).

In combination with other ubiquitin ligases, Ubr1 also can ubiquitylate internally misfolded proteins. For instance, deletions of both *UBR1* and *SAN1* are necessary to fully stabilize several engineered cytosolic substrates (*e.g.*, ΔssCPY^*^-GFP derived from the misfolded vacuolar carboxypeptidase Y mutant) (Heck *et al.* 2010; Prasad *et al.* 2010). Furthermore, Ubr1 was shown to target newly synthesized cytosolic unfolded protein kinases together with the closely related Ubr2 E3 ligase (Nillegoda *et al.* 2010). In addition to Ubr1, other ubiquitin ligases have been implicated in targeting yeast cytosolic proteins. The endoplasmic reticulum−associated degradation Doa10 ubiquitin ligase was shown to target temperature-sensitive alleles of Ura3 (Lewis and Pelham 2009), as well as Ura3 fused to the CL1 degron (Metzger *et al.* 2008; Ravid *et al.* 2006). More recently, the Rkr1/Ltn1 E3 ligase was found to associate with ribosomes and to target nascent nonstop polypeptides that are stalled during translation for degradation (Bengtson and Joazeiro 2010). In addition, the HECT ubiquitin ligase Hul5 was identified as a major player in the ubiquitylation of low-solubility cytosolic proteins after heat-shock stress and in physiological conditions. Deletion of *HUL5* reduced growth after heat-shock and decreased the degradation of short-lived misfolded proteins in the cell (Fang *et al.* 2011). Given the number of ubiquitin ligases that target cytosolic misfolded proteins, it is unknown how broad their respective spectrum of substrates is.

Protein QC pathways have classically been elucidated using mutant model substrates in yeast (Jung *et al.* 1999; McClellan *et al.* 2005; Wolf and Schafer 2005). It was previously reported that the thermosensitive (*ts*) nuclear mutant Ubc9-1 was short-lived and targeted for degradation by the proteasome at the restrictive temperature (Betting and Seufert 1996). Similarly, a *ts* mutant of Ubc4, with a single point mutation, was also found to be rapidly degraded at the nonpermissive temperature (Tongaonkar *et al.* 1999). Several other *ts* mutants were subsequently found to be unstable, including the nuclear proteins Cdc68-1, Sir4-9, Cdc13-1, Sir3-8, and the ER protein Mp2-1 (Gardner *et al.* 2005; Ravid *et al.* 2006). Deletion of the E3 ligases *SAN1* and *DOA10* leads to significant stabilization of *cdc68-1* and of *msp2-1* mutants, respectively, and restoration of viability under restrictive conditions (Gardner *et al.* 2005; Ravid *et al.* 2006). This result suggests that the *ts* lethal phenotype of these essential gene mutants is conditional to the degradation of the mutated proteins at a higher temperature. Less clear is whether the same phenomenon applies to cytosolic misfolded proteins and the extent to which lethality of *ts* mutants depends on proteolysis.

In this report, we have analyzed a panel of *ts* alleles corresponding to different cytosolic proteins and observed a variable stability profile for the different mutants. We further characterized the unstable alleles and found that the Ubr1 pathway plays a major role in the degradation of these misfolded proteins.

## Materials and Methods

### Yeast strains and plasmids

Media preparation and molecular biology techniques were performed using standard procedures. Yeast strains used in this study are outline in supporting information, Table S1. The *ts* alleles used in this study were generously provided by Phil Hieter and were previously obtained by random polymerase chain reaction mutagenesis of essential genes that were then integrated in their endogenous loci after selection for conditional *ts* lethality (Ben-Aroya *et al.* 2008, 2010). In this study, the yeast temperature-sensitive strains were tagged C-terminally by homologous recombination using 13MYC-KanMX6 module (Longtine *et al.* 1998). The deletions were generated by directly knocking out the specific gene by homologous recombination (Rothstein 1991) or by crossing with cells carrying single gene deletion (c*an1Δ*::*STE2pr-spHIS5*, *lyp1Δ*, *his3Δ1*, *leu2Δ0*, *ura3Δ0*, *met15Δ0*, *geneX*::*NatMX4*) (Baryshnikova *et al.* 2010; Tong and Boone 2006) followed by tetrad dissection.

### Immunochemistry

Protein degradation was examined by cycloheximide chase assays. Cells were grown in YPD to exponential phase (OD_600_ = 1) at permissive temperature (25°) before the addition of cycloheximide (100 µg/mL). The cells were then either incubated at the permissive temperature or restrictive temperature (37°). For proteasome inhibition experiments, cells were grown to exponential phase in synthetic complete medium (0.17% yeast nitrogen base without ammonium sulfate) supplemented with 0.1% proline and 2% glucose as the carbon source (Liu *et al.* 2007). The overnight grown culture was diluted with fresh media supplemented with 0.003% SDS at OD_600_ 0.2. The cells were treated with 20 µM MG132 or control dimethyl sulfoxide (DMSO) for 30 min and then with 100 µg/mL cycloheximide. Samples were collected at indicated time points and lysed with a modified Laemmli lysis buffer (50 mM Tris-HCl pH 6.8, 2% sodium dodecyl sulfate [SDS], 10% glycerol) with glass beads. Protein concentration was determined by DC-Protein assay (Bio-Rad). Equal amounts of protein were resolved by SDS polyacrylamide gel electrophoresis (PAGE). To assess solubility, cells were lysed with glass beads in a native lysis buffer [0.5% NP-40, 20 mM HEPES, 200 mM NaCl, 1 mM ethylene diamine tetraacetic acid, protease inhibitors (Roche Applied Science), 1 mM phenanthroline]. The lysates were first precleared by centrifugation in a microfuge (2000 *g*, 5 min at 4°), then further fractionated to soluble and insoluble fractions by centrifugation (16,000 *g*, 10 min, 4°). Immunoblots were performed with 9E10 monoclonal mouse antibody (1:7000 dilution) against MYC-tagged proteins, and anti-Pgk1 monoclonal rabbit antibody as loading control (1:10,000 dilution; gift from J. Thorner, University of California). Antimouse and antirabbit secondary antibodies, coupled to Cy3 and Cy5, respectively (Mandel Scientific, 1:10,000), were used. Protein bands were quantified by using Li-Cor Odyssey Fluorescent detection system.

### Structure prediction

MODELER (Sali and Blundell 1993; Shen and Sali 2006) was used to generate models of Pro3 using homologs from several different organisms as templates (PDB codes 1YQG, 2AHR, 2IZZ, 2RCY). First a structurally influenced alignment of the templates was generated using the salign function, and then the Pro3 sequence was appended and aligned. A total of 100 models were generated on the basis of this alignment and scored using the DOPE statistical potential. The best-scoring model was used to fit mutated residues. To generate a model of the dimer, two copies of the best-scoring Pro3 model were structurally aligned (using PyMol) to chains A & E of PDB structure 2RCY, the most closely related homolog. Models of Pro3-1 and Pro3-2 were generated in the same way, by replacing the relevant amino acids in the alignment.

## Results

### A large portion of *ts* mutants is degraded at restrictive temperature

We reasoned that a large fraction of *ts* mutants might be unstable at restrictive temperature. We selected from a mutant library generated by polymerase chain reaction mutagenesis (Ben-Aroya *et al.* 2008) a panel of 22 *ts* alleles of six essential genes encoding for primarily cytosolic proteins: *PRO3*, *GUS1*, *GUK1*, *GLN1*, *UGP1*, and *GRS1*. Delta 1-pyrroline carboxylate reductase (*PRO3*) is a multimeric enzyme involved in the conversion of delta-1-pyrroline carboxylate reductase to proline in the cytosol (Brandriss and Falvey 1992). Glutamyl tRNA synthetase (*GUS1*) and Glycyl tRNA synthetase (*GRS1*) are cytosolic enzymes that ligate amino acids to cognate tRNA (Delarue 1995; Galani *et al.* 2001). Glutamine synthetase (*GLN1*) is a metabolic enzyme in the cytosol that catalyzes amination of glutamate to form glutamine (Benjamin *et al.* 1989). Guanylate kinase (*GUK1*), localized both in the cytosol and nucleus, converts GMP to GDP and is required for mannose chain elongation in the eukaryotic cell wall (Shimma *et al.* 1997). UDP-glucose pyrophosphorylate (*UGP1*) is a cytosolic enzyme involved in the formation of UDP-glucose from glucose-1-phosphate and UTP (Lai and Elsas 2000). All these proteins were estimated to be present in high abundance in the cell (>10,000 copies/ cell) and relatively stable (t_1/2_ > 40−300 min) (Belle *et al.* 2006).

**Figure 1:**
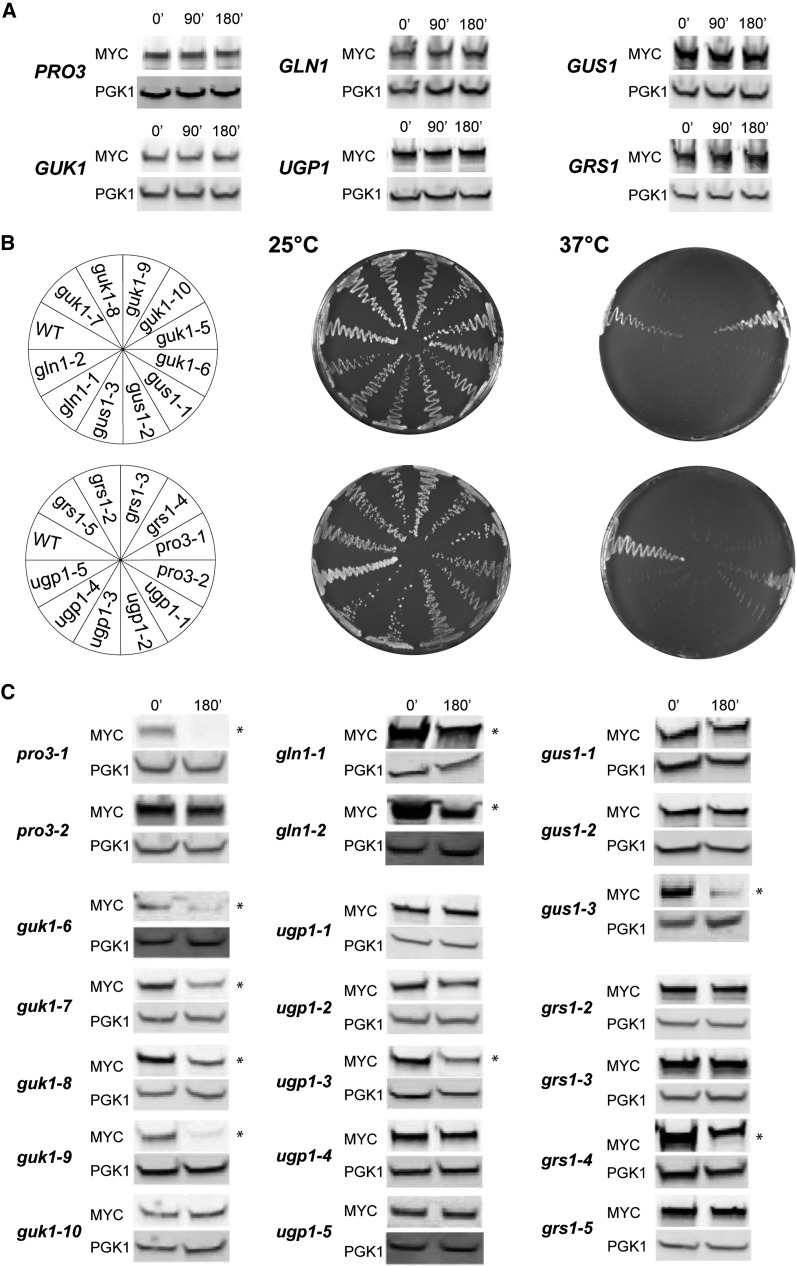
A large fraction of *ts* mutant proteins are degraded at restrictive temperature. (A) The stability of wild-type cytosolic proteins was assessed at 37° after the addition of cycloheximide to cells grown at 25°. Cells were collected at the indicated time (min), and equal amount of proteins were analyzed by Western blotting using anti-MYC 9E10 antibody and anti-Pgk1 antibodies. (B) Yeast strains carrying the indicated C-terminally tagged *ts* alleles, and wild-type BY4741 cells were streaked on synthetic complete media, and viability was determined after three days of growth at the indicated temperatures. (C) The stability of mutant proteins was determined after a 3-hr cycloheximide treatment at 37° as in (A). Asterisks denote alleles that displayed more than a 50% protein level decrease.

To assess the stability of the selected proteins, wild-type and *ts* alleles were C-terminally tagged with 13 MYC epitopes. We observed that the tested wild-type alleles are fully stable after the addition of the translation inhibitor cycloheximide to cells growing at 37° for 3 hr (Figure 1A). We then assessed the viability of the C-terminally tagged mutant alleles on synthetic complete media at permissive (25°) and restrictive temperatures (37°). We found that 19 of the 22 strains maintained a complete loss of viability phenotype at restrictive temperature, two strains displayed a subdued phenotype (*pro3-2* and *ugp1-1*), and one strain was fully viable (*guk1-5*; Figure 1B). We confirmed that the untagged *guk1-5* mutant remained *ts* at restrictive temperature in our growth conditions (data not shown). We reasoned that the addition of the C-terminal tag rescues the *ts* phenotype of *guk1-5* (which was not further characterized) while not affecting the temperature sensitivity of most tagged alleles.

We next assessed the stability of the tagged *ts* mutant proteins at restrictive temperature. We found that 10 of the 21 assessed proteins displayed 50% or greater reduction in protein levels after the addition of cycloheximide to cells growing at restrictive temperature for 3 hr (Figure 1C). Distinct stability was observed for different alleles of the same protein, as for the guanylate kinase (*GUK1*). Two alleles (Guk1-6 and Guk1-9) were almost completely degraded, two other alleles displayed substantial reduction (Guk1-7, Guk1-8), whereas another *ts* allele was stable (Guk1-10). Overall, we observed that approximately one-half of the *ts* mutants displayed greater protein turnover at the restrictive temperature. These results suggest that a large portion of yeast *ts* alleles encodes for unstable proteins.

### The *ts* mutant Pro3-1 is preferentially degraded at restrictive temperature

**Figure 2:**
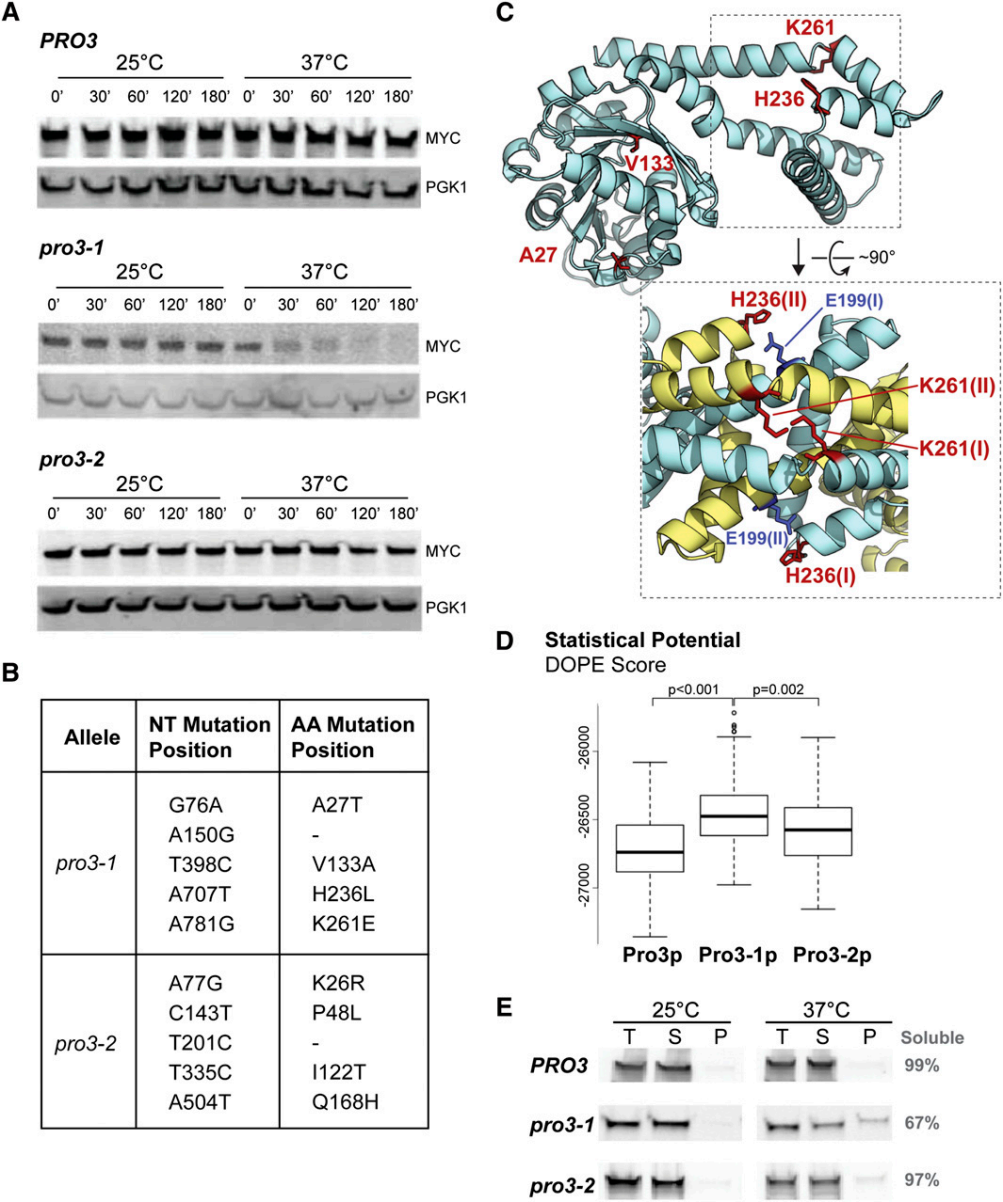
*PRO3* alleles display variable stability and solubility. (A) Wild-type *PRO3* and mutant strains *pro3-1* and *pro3-2* were grown to exponential phase, and the stability of the protein was assessed over a 3-hr time course after the addition of cycloheximide-chase at 25° and 37°. Western blot analysis was performed with 9E10 and anti-Pgk1 antibodies. (B) The mutant alleles of *PRO3* were amplified via polymerase chain reaction from their endogenous locus and sequenced to determine number and location of mutations. Positions and substitutions are indicated for the mutated nucleotides and amino acids. (C) Model of Pro3 showing residues mutated in Pro3-1. Residues that are mutated in Pro3-1 are shown in red stick representations. Two are found in the Rossmann fold domain, and two are in the dimerization domain. The inset view shows a model of the dimer, with the relevant residues shown in stick representations. One unit of the Pro3 dimer is depicted in blue (I) and the second unit in yellow (II). Lysine 261 residues from each subunit come into close contact. These are mutated to glutamic acid in Pro3-1. There is also the potential for inter-subunit contact between histidine 236 (mutated to leucine in Pro3-1) and glutamic acid 199 of the opposing subunit. (D) Boxplot of statistical potentials of Pro3 models. The statistical potential (DOPE) scores for all Pro3 models are compared. *P* values were derived using the Wilcoxon test. Pro3-1 models show the highest potential, indicating that this sequence generates more unsuitable models. This implies that Pro3-1 mutations likely destabilize the structure. (E) The solubility of wild-type (Pro3) and mutants (Pro3-1 and Pro3-2) was assessed before and after shifting the cells from 25° to 37° for 30 min. An equal portion of each fraction (T, total cell lysate; S, supernatant; P, recovered from pellet) was loaded on SDS-PAGE for western blot analysis with 9E10 antibody. The portion of the recovered signal in the soluble fraction is indicated (gray).

We next sought to examine the turnover rate of the Pro3-1 mutant in more detail because of its pronounced degradation in the first assay. We confirmed that Pro3-1 is efficiently degraded over a 3-hr cycloheximide incubation at the nonpermissive temperature, whereas it is fully stable at the permissive temperature (Figure 2A). In contrast, wild-type Pro3 is completely stable at both the permissive and restrictive temperatures during the same 3-hr chase period. Similarly, we found that the second mutant, Pro3-2, was also fully stable at both the permissive and nonpermissive temperatures (Figure 2A). Interestingly, this mutant displayed a subdued *ts* phenotype, which might be explained by the greater stability of the protein at 37° as compared with Pro3-1.

Upon sequencing the mutant alleles, we found that both *pro3-1* and *pro3-2* mutant alleles each have four missense mutations and one silent mutation in their open-reading frames (Figure 2B). We next mapped the mutations of Pro3-1 on a homology structure model. It has been shown that Pro3 is a multimer in higher eukaryotes (Meng *et al.* 2006). Two of the mutated residues (H236, K261) are found near the binding interface involved in Pro3 homo-dimerization and the two other sites (A27, V133) are located within the catalytic domain of Pro3 (Figure 2C). Interestingly, the lysine (K261) residue of Pro3 is closely located to its counterpart in the second homo-dimer unit (inset in Figure 2C). In addition, the histidine (H236) is located near a negatively charged glutamic acid (E199) of the opposing subunit. These data indicates that both residues may be important for the formation of the Pro3 homo-dimer. One possibility is that these two mutated residues may affect dimerzation and stability of Pro3-1. In agreement with this notion, the DOPE (*i.e.*, discrete optimized protein energy) (Shen and Sali 2006) scores of the homology model structures generated for Pro3-1 were significantly greater compared with wild-type Pro3 and Pro3-2 (Figure 2D), further indicating that the conformation of this mutant may be less stable. To test this idea, we examined the solubility of the three alleles of Pro3 after centrifugation at the nonpermissive temperature. We found that after a 30-min incubation period at 37°, a significant portion of Pro3-1 (>30%) is deposited in the insoluble fraction (Figure 2E). In contrast, only minute portions of Pro3-2 and of the wild-type Pro3 are insoluble in these conditions. These results indicate that, upon shifting the cells to the restrictive temperature, a large portion of Pro3-1 likely misfolds and aggregates.

### The loss of stability of the *ts* mutants at restrictive temperature is dependent on the proteasome

**Figure 3:**
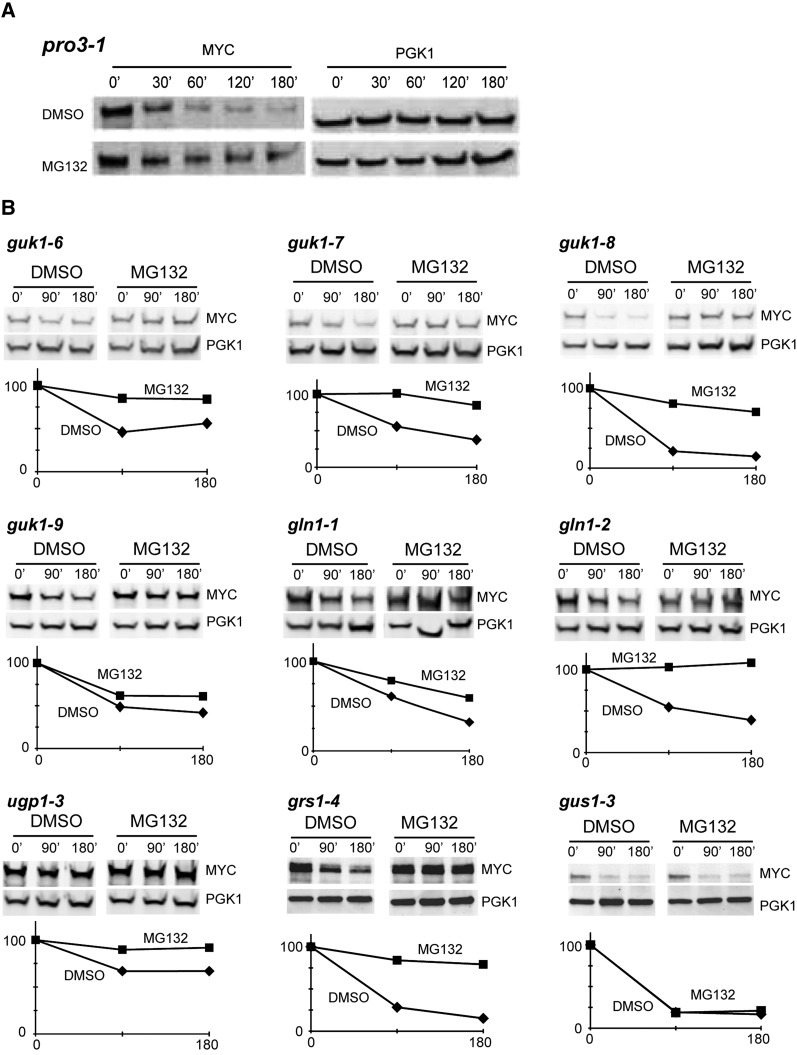
The degradation of *ts* mutants is dependent on the proteasome. (A) Mutant strain *pro3-1* was grown to exponential phase and preincubated for 30 min with either dimethyl sulfoxide or proteasome inhibitor MG132 at 25°, then shifted to 37° with cycloheximide. The stability of Pro3-1, at the indicated time points (min), was determined by western blot analysis using 9E10 and anti-Pgk1 antibodies. (B) Mutant strains of unstable *ts* alleles were treated as in (A). For each allele, the graph represents the relative protein levels normalized with Pgk1 signal that were quantified at the indicated time points (min).

We postulated that the degradation of the Pro3-1 mutant is mediated by a proteasome-dependent QC pathway. Incubation of *pro3-1* temperature-sensitive cells with the proteasome inhibitor MG132 impaired the rapid turnover of the mutant protein after shifting the cells at 37° (Figure 3A). This finding indicates that degradation of Pro3-1 is, at least partially, dependent on the proteasome.

We next examined whether the other nine unstable *ts* mutants are also targeted to the proteasome. A large subset of the unstable mutant proteins is completely stabilized by proteasome inhibition (Figure 3B), whereas two others are only partially stabilized (*gln1-1* and *guk1-9*) and one *ts* allele degradation is not dependent on the proteasome (*gus1-3)*. This finding suggests that the proteolysis of most unstable *ts* mutants is mediated by the proteasome, whereas a few other mutants are degraded by a different pathway in the cell (*e.g.* macro-autophagy).

### Double deletion of *UBR1* and *SAN1* significantly stabilizes Pro3-1 and suppresses lethality of *pro3-1*

**Figure 4:**
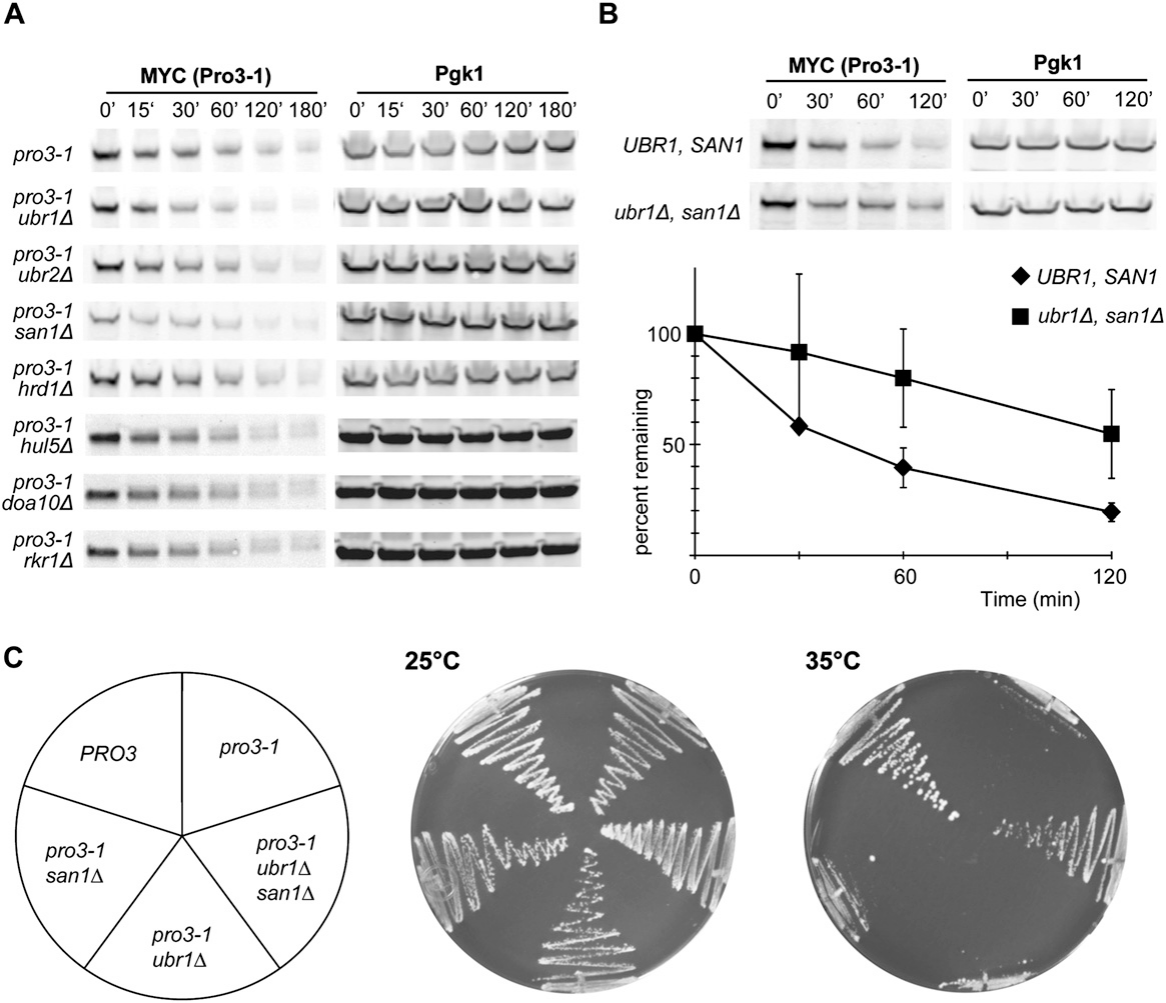
Deletions of *UBR1* and *SAN1* stabilize Pro3-1 and suppress the *ts* phenotype. (A) Haploids *pro3-1* strains with the indicated single deletions were grown to exponential phase at 25°, and the stability of Pro3-1 was assessed after the addition of cycloheximide at the indicated time points over a 3-hr period incubation at 37°. Levels of Pro3-1 were determined by western blot analysis using 9E10 and anti-Pgk1 antibodies. (B) Levels of pro3-1 in *SAN1*, *UBR1*, and *san1Δ ubr1Δ* cells were assessed as in (A). The graph represents relative levels of Pro3-1, normalized with Pgk1 signal, that were quantified at the indicated time points (min) and averaged from three independent experiments (with standard deviations). (C) Viability of haploids *pro3-1* strain with the indicated deletions was assessed on plates with synthetic complete media at 25° and 35° for 3 days.

We next sought to determine which ubiquitin ligase involved in a QC pathway may be required for targeting Pro3-1 for degradation by the proteasome. We directly assessed the stability of Pro3-1 in cells containing single deletion of *SAN1*, *UBR1*, *UBR2*, *HRD1*, *DOA10*, *RKR1*, and *HUL5*, which are the major yeast ubiquitin ligases involved in the degradation of misfolded proteins. We found that none of the tested single deletions had a significant effect on the stability of Pro3-1 when the cells were shifted to the restrictive temperature along with addition of cycloheximide (Figure 4A). In contrast, we found that there was a significant reduction of the turnover of Pro3-1 in the absence of both Ubr1 and San1 E3 ligases in comparison with control cells (Figure 4B). This finding indicates that Ubr1 and San1 together play a major role in targeting of Pro3-1 for degradation.

We then determined whether *pro3-1* lethality could be rescued by a reduced turnover of the mutant protein. We assessed the viability of different *pro3-1* strains with single and double deletions of *UBR1* and *SAN1*. We found that the single deletions of *UBR1* and *SAN1* did not suppress the *ts* phenotype, whereas the double ligase deletion restored the growth at the nonpermissive temperature (Figure 4C). This finding suggests that the conditional lethality of *pro3-1* is most likely attributable to the QC degradation of the misfolded polypeptide, leading to depletion of the essential protein at restrictive temperature.

### Ubr1 is an important constituent of the cytosolic QC machinery

**Figure 5:**
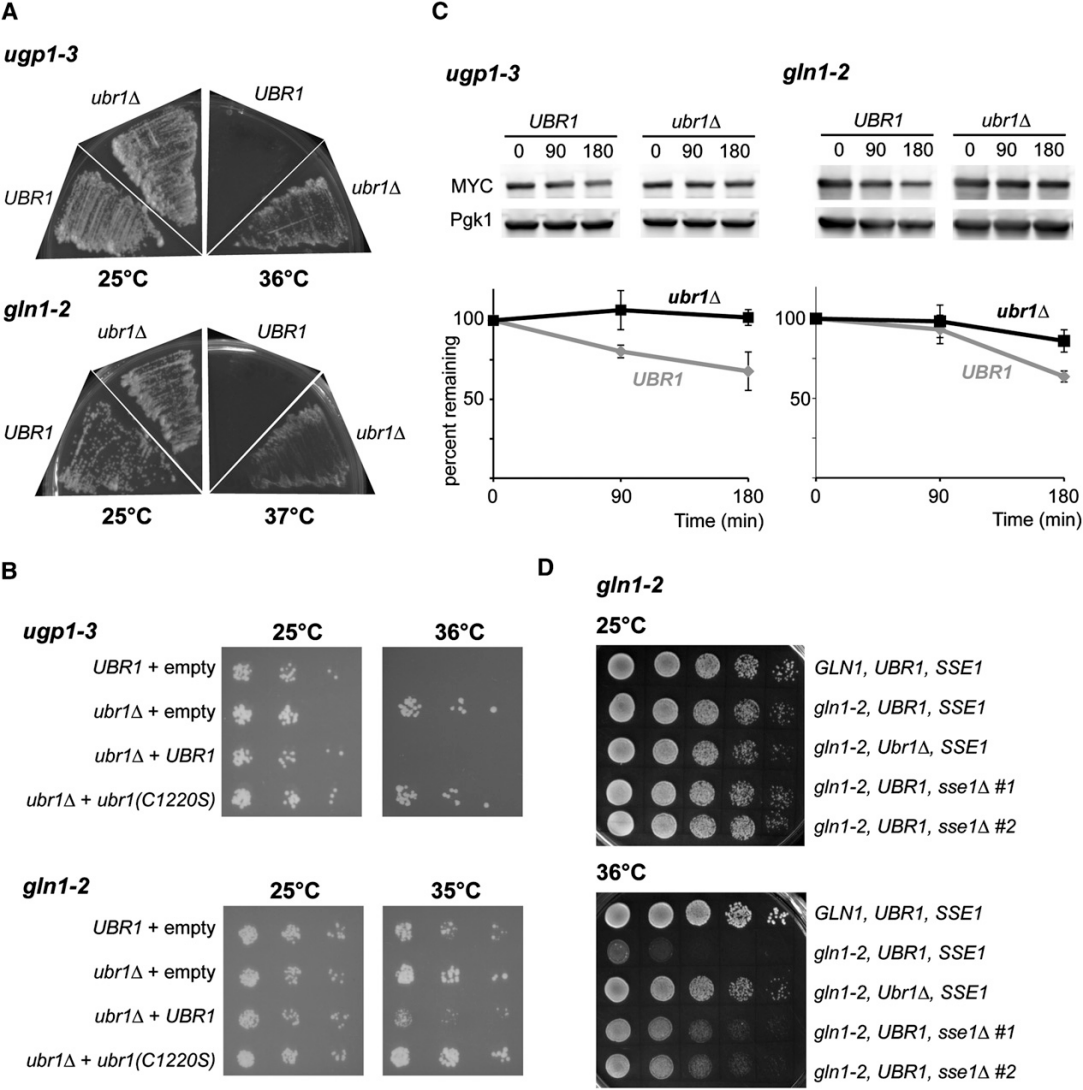
The Ubr1 ubiquitin ligase is required for the degradation of Ugp1-3 and Gln1-2. (A) Viability of *ugp1-3* and *gln1-2* haploid strains (*UBR1* or *ubr1Δ*) was assessed on plates with yeast peptone dextrose at the indicated temperatures. (B) Viability of *ugp1-3* and *gln1-2* haploid strains (*UBR1* or *ubr1Δ*) with an empty plasmid (pRS315) or expressing wild-type or inactive RING mutant Ubr1 were assessed on SD-LEU plates at the indicated temperatures after 1/5 dilutions of the cell cultures. (C) Levels of Ugp1-3 and Gln1-2 in *UBR1* and *ubr1Δ* cells were assessed at 37° after the addition of cycloheximide to cells grown at 25° (OD_600_ ~1). Levels of the MYC-tagged mutant proteins were determined by western blot analysis using 9E10 and anti-Pgk1 antibodies. The graph represents relative levels, normalized with Pgk1 signal, quantified at the indicated time points and averaged from three independent experiments (with standard deviations). (D) Viability of *gln1-2* haploid strains (with *UBR1* or *SSE1* deletions) was assessed on plates at the indicated temperatures. Two different sse1Δ strains were assessed.

We then sought to determine whether the other *ts* alleles degraded in a proteasome-dependent manner (partially or fully) are affected by the absence of Ubr1. We found that lethality of both *ugp1-3* and *gln1-2* is restored by *UBR1* deletion (Figure 5A). Because single deletion of *UBR1* rescues the loss of function, we performed add-back experiments with either wild-type or inactive RING mutant (C1220S) Ubr1 ubiquitin ligase. Lethality was restored in presence of the wild-type but not of the inactive ligase, further indicating that the phenotype was dependent on the ubiquitin ligase activity of Ubr1 (Figure 5B). Double deletion of both *UBR1* and *SAN1* did not suppress lethality of the other alleles, nor did it further augment the recovery of *ugp1-3* and *gln1-2* (data not shown). These data suggest that, in some cases, targeting of QC substrates for degradation is mainly mediated by the Ubr1 E3 ligase.

We next assessed the turnover of *ugp1-3* and *gln1*-2 mutant proteins at restrictive temperature in the absence of the Ubr1 ubiquitin ligase. In both instances, deletion of *UBR1* caused stabilization of the mutant proteins after shifting the cells at the nonpermissive temperature in the presence of cycloheximide (Figure 5C). Overall, these results indicate that *UBR1* is involved in the proteasomal degradation of a substantial number (3/9) of unstable *ts* alleles of cytosolic proteins.

To gain further understanding of the targeting of the mutant proteins by Ubr1, we tested whether the deletion of *SSE1*, encoding for the Hsp70 nucleotide exchange factor, could also rescue the lethality of the *ts* allele. It was previously shown that degradation of the VHL model substrate in yeast requires *SSE1* (McClellan *et al.* 2005). Similarly, the degradation of three engineered Ubr1 substrates required *SSE1* (Heck *et al.* 2010; Prasad *et al.* 2010). In addition, *in vitro* ubiquitylation of tGnd1-GFP (truncation fragment of Gdn1 fused to GFP) was dramatically reduced when using extracts derived from *sse1Δ* cells (Heck *et al.* 2010), indicating that Sse1 may mediate or enhance the recognition of the misfolded substrate by Ubr1. We therefore tested whether deletion of *SSE1* could rescue the lethality of *pro3-1*, *ugp1-3*, and *gln1*-2. We found that lethality of *gln1*-2 was partially rescued in *sse1Δ* cells (Figure 5D), whereas no effect was observed for *pro3-1* and *ugp1-3* at different nonpermissive temperatures (35, 36, or 37°; data not shown). These data indicates that *SSE1* may only be involved in the degradation of a subset of Ubr1-misfolded substrates.

## Discussion

Degradative QC pathways have been classically elucidated using model protein substrates. In this study, we show that *ts* mutants of cytosolic proteins display key features of QC substrates that can then be used to characterize the cytosolic QC machinery. Using this panel of model substrates, we found that the ubiquitin ligase Ubr1 plays a major role in targeting cytosolic *ts* substrates for degradation.

In most cases, *ts* mutants arise by mutations in essential genes that lead to a loss of viability under restrictive conditions. These mutations may impair protein function by altering the catalytic activity, the propensity to bind to physiological partners, as well as by affecting steady-state levels (by modifying protein expression or stability). We hypothesized that missense mutations that lead to conditional lethality may affect the steady-state levels of a large portion of essential proteins by inducing misfolding and degradation. We observed an increased protein turnover for approximately half the tested alleles under restrictive conditions (10/21), suggesting that reduced protein half-life may be a major cause of the *ts* phenotype. Importantly, each time the degradation of a *ts* mutant was reduced by genetic manipulations, the viability of the cell at nonpermissive temperature was restored.

In most cases, the turnover of these unstable *ts* mutant is dependent (fully or partially) on the proteasome activity, indicating that the ubiquitin proteasome system plays a key role in targeting these mutant proteins. Interestingly, degradation of these normally long-lived proteins occurs presumably after folding to their native state at permissive temperature. In contrast to the turnover of newly synthesized proteins after translation (typically degraded within a few minutes), the half-life of these mutants at nonpermissive temperature was relatively long (>30 min). Hence, the *ts* phenotype of these alleles may arise from a relatively slow decay of the essential proteins.

These *ts* alleles of cytosolic proteins constitute a new class of model substrates to study QC. So far, mainly ectopic artificial substrates, such as truncated carboxypeptidase Y or GFP, as well as general misfolding stresses, have been used to elucidate yeast protein QC pathways in the cytoplasm (Fang *et al.* 2011; Heck *et al.* 2010; Nillegoda *et al.* 2010; Prasad *et al.* 2010). The use of these approaches does not always fully mimic the physiological conditions or encapsulate the full spectrum of substrates targeted by protein QC. The panel of *ts* substrates used in this study are advantageous because they are expressed under the endogenous promoters. We found that as few as two missense mutations can lead to the decreased stability of the *ts* mutants guk1-6 and guk1-8 (data not shown). Misfolding and degradation can be induced by shifting cells to nonpermissive temperature without inducing a full stress response. Thus, interactions with these *ts* model substrates are likely pertinent to physiological conditions and potentially relevant to human autosomal recessive genetic diseases caused by protein misfolding.

We next sought to determine how prominent the role of Ubr1 is in cytosolic QC by using the panel of cytosolic *ts* mutants. To date, six of seven E3 ligases involved in protein QC have been either exclusively or partially implicated in cytosolic QC in *Saccharomyces cerevisiae* (Bengtson and Joazeiro 2010; Fang *et al.* 2011; Gardner *et al.* 2005; Heck *et al.* 2010; Lewis and Pelham 2009; Nillegoda *et al.* 2010; Prasad *et al.* 2010; Ravid *et al.* 2006), and it is not clear which is (or are) the primary pathway(s) in the cell. We found that Ubr1 plays a substantial role in cytosolic QC, as the proteasome degradation of three of nine cytosolic mutant proteins was significantly reduced by *UBR1* deletion. In two other cases (*guk1-6* and *guk1-8*), we also observed a recovery in some—but not all—cells, which may be attributable to the reversion of the phenotype by additional mutations (data not shown). In one case (*pro3-1*), only deletion of both *SAN1* and *UBR1* leads to a significant stabilization of the mutant, indicating that both ubiquitin ligases are redundant. The same observation was made in previous detailed analyses of artificial model substrates (Heck *et al.* 2010; Prasad *et al.* 2010). Intriguingly, the authors of previous studies have shown that *SSE1* was important for the degradation of Ubr1 substrates. In our hands, only *gln1-2* was partially rescued by *SSE1* deletion. One possibility is that by increasing the temperature, several chaperone proteins may be induced and compensate for the absence of Sse1. Interestingly, San1 was shown to interact with the truncated version of Pro3 by yeast two-hybrid assay (Rosenbaum *et al.* 2011). The targeting of cytosolic proteins by San1 ligase may depend on their propensity to bind to San1 and/or to shuttle to the nucleus. Note that as the degradation of Pro3-1 was only partially blocked by proteasome inhibition, it suggests that another proteasome-independent pathway is also involved in the turnover of that mutant.

It remains unclear which pathways target the other unstable mutants. Single deletions of Doa10 and Hul5, as well double deletion of San1 and Ubr1, do not rescue the lethality of the other *ts* alleles (data not shown). One possibility is that some of the unstable proteins are ubiquitylated by several redundant ubiquitin ligases (*e.g.*, Ubr1 and Doa10); alternatively, these mutants could also be targeted by another, not yet identified, QC E3. Future studies will help to delineate these degradative QC pathways further.

## Supplementary Material

Supporting Information

## References

[bib1] Anelli T. Sitia R., 2008 Protein quality control in the early secretory pathway. EMBO J. 27: 315–327 18216874 10.1038/sj.emboj.7601974 PMC2234347

[bib2] Bartel B. Wunning I. Varshavsky A., 1990 The recognition component of the N-end rule pathway. EMBO J. 9: 3179–3189 2209542 10.1002/j.1460-2075.1990.tb07516.x PMC552047

[bib3] Baryshnikova A. Costanzo M. Dixon S. Vizeacoumar F. J. Myers C. L., 2010 Synthetic genetic array (SGA) analysis in *Saccharomyces cerevisiae* and schizosaccharomyces pombe. Methods Enzymol. 470: 145–179 20946810 10.1016/S0076-6879(10)70007-0

[bib4] Belle A. Tanay A. Bitincka L. Shamir R. O’Shea E. K., 2006 Quantification of protein half-lives in the budding yeast proteome. Proc. Natl. Acad. Sci. USA 103: 13004–13009 16916930 10.1073/pnas.0605420103 PMC1550773

[bib5] Ben-Aroya S. Coombes C. Kwok T. O’Donnell K. A. Boeke J. D., 2008 Toward a comprehensive temperature-sensitive mutant repository of the essential genes of saccharomyces cerevisiae. Mol. Cell 30: 248–258 18439903 10.1016/j.molcel.2008.02.021 PMC4130347

[bib6] Ben-Aroya S. Pan X. Boeke J. D. Hieter P., 2010 Making temperature-sensitive mutants. Methods Enzymol. 470: 181–204 20946811 10.1016/S0076-6879(10)70008-2 PMC2957654

[bib7] Bengtson M. H. Joazeiro C. A., 2010 Role of a ribosome-associated E3 ubiquitin ligase in protein quality control. Nature 467: 470–473 20835226 10.1038/nature09371 PMC2988496

[bib8] Benjamin P. M. Wu J. I. Mitchell A. P. Magasanik B., 1989 Three regulatory systems control expression of glutamine synthetase in saccharomyces cerevisiae at the level of transcription. Mol. Gen. Genet. 217: 370–377 2570348 10.1007/BF02464906

[bib9] Betting J. Seufert W., 1996 A yeast Ubc9 mutant protein with temperature-sensitive in vivo function is subject to conditional proteolysis by a ubiquitin- and proteasome-dependent pathway. J. Biol. Chem. 271: 25790–25796 8824207 10.1074/jbc.271.42.25790

[bib10] Brandriss M. C. Falvey D. A., 1992 Proline biosynthesis in saccharomyces cerevisiae: Analysis of the PRO3 gene, which encodes delta 1-pyrroline-5-carboxylate reductase. J. Bacteriol. 174: 5176 1352771 10.1128/jb.174.15.5176b.1992 PMC206344

[bib11] Buchberger A. Bukau B. Sommer T., 2010 Protein quality control in the cytosol and the endoplasmic reticulum: Brothers in arms. Mol. Cell 40: 238–252 20965419 10.1016/j.molcel.2010.10.001

[bib12] Cheng S. H. Gregory R. J. Marshall J. Paul S. Souza D. W., 1990 Defective intracellular transport and processing of CFTR is the molecular basis of most cystic fibrosis. Cell 63: 827–834 1699669 10.1016/0092-8674(90)90148-8

[bib13] Connell P. Ballinger C. A. Jiang J. Wu Y. Thompson L. J., 2001 The co-chaperone CHIP regulates protein triage decisions mediated by heat-shock proteins. Nat. Cell Biol. 3: 93–96 11146632 10.1038/35050618

[bib14] Delarue M., 1995 Aminoacyl-tRNA synthetases. Curr. Opin. Struct. Biol. 5: 48–55 7773747 10.1016/0959-440x(95)80008-o

[bib15] Eisele F. Wolf D. H., 2008 Degradation of misfolded protein in the cytoplasm is mediated by the ubiquitin ligase Ubr1. FEBS Lett. 582: 4143–4146 19041308 10.1016/j.febslet.2008.11.015

[bib16] Fang N. N. Ng A. H. Measday V. Mayor T., 2011 Hul5 HECT ubiquitin ligase plays a major role in the ubiquitylation and turnover of cytosolic misfolded proteins. Nat. Cell Biol. 13: 1344–1352 21983566 10.1038/ncb2343 PMC4961474

[bib17] Finley D., 2009 Recognition and processing of ubiquitin-protein conjugates by the proteasome. Annu. Rev. Biochem. 78: 477–513 19489727 10.1146/annurev.biochem.78.081507.101607 PMC3431160

[bib18] Galani K. Grosshans H. Deinert K. Hurt E. C. Simos G., 2001 The intracellular location of two aminoacyl-tRNA synthetases depends on complex formation with Arc1p. EMBO J. 20: 6889–6898 11726524 10.1093/emboj/20.23.6889 PMC125769

[bib19] Gardner R. G. Nelson Z. W. Gottschling D. E., 2005 Degradation-mediated protein quality control in the nucleus. Cell 120: 803–815 15797381 10.1016/j.cell.2005.01.016

[bib20] Heck J. W. Cheung S. K. Hampton R. Y., 2010 Cytoplasmic protein quality control degradation mediated by parallel actions of the E3 ubiquitin ligases Ubr1 and San1. Proc. Natl. Acad. Sci. USA 107: 1106–1111 20080635 10.1073/pnas.0910591107 PMC2824284

[bib21] Hirsch C. Gauss R. Horn S. C. Neuber O. Sommer T., 2009 The ubiquitylation machinery of the endoplasmic reticulum. Nature 458: 453–460 19325625 10.1038/nature07962

[bib22] Hol E. M. Scheper W., 2008 Protein quality control in neurodegeneration: Walking the tight rope between health and disease. J. Mol. Neurosci. 34: 23–33 18157655 10.1007/s12031-007-0013-8

[bib23] Huh W. K. Falvo J. V. Gerke L. C. Carroll A. S. Howson R. W., 2003 Global analysis of protein localization in budding yeast. Nature 425: 686–691 14562095 10.1038/nature02026

[bib24] Jung G. Ueno H. Hayashi R., 1999 Carboxypeptidase Y: Structural basis for protein sorting and catalytic triad. J. Biochem. 126: 1–6 10393313 10.1093/oxfordjournals.jbchem.a022408

[bib25] Lai K. Elsas L. J., 2000 Overexpression of human UDP-glucose pyrophosphorylase rescues galactose-1-phosphate uridyltransferase-deficient yeast. Biochem. Biophys. Res. Commun. 271: 392–400 10799308 10.1006/bbrc.2000.2629

[bib26] Lewis M. J. Pelham H. R., 2009 Inefficient quality control of thermosensitive proteins on the plasma membrane. PLoS ONE 4: e5038 19337370 10.1371/journal.pone.0005038 PMC2659772

[bib27] Lichter-Konecki U. Konecki D. S. DiLella A. G. Brayton K. Marvit J., 1988 Phenylalanine hydroxylase deficiency caused by a single base substitution in an exon of the human phenylalanine hydroxylase gene. Biochemistry 27: 2881–2885 2840952 10.1021/bi00408a032

[bib28] Liu C. Apodaca J. Davis L. E. Rao H., 2007 Proteasome inhibition in wild-type yeast *Saccharomyces cerevisiae* cells. Biotechniques 42: 158, 160, 162 17373478 10.2144/000112389

[bib29] Longtine M. S. McKenzie A. 3rd Demarini D. J. Shah N. G. Wach A., 1998 Additional modules for versatile and economical PCR-based gene deletion and modification in *Saccharomyces cerevisiae*. Yeast 14: 953–961 9717241 10.1002/(SICI)1097-0061(199807)14:10<953::AID-YEA293>3.0.CO;2-U

[bib30] Matsuo Y. Kishimoto H. Tanae K. Kitamura K. Katayama S., 2011 Nuclear protein quality is regulated by the ubiquitin-proteasome system through the activity of Ubc4 and San1 in fission yeast. J. Biol. Chem. 286: 13775–13790 21324894 10.1074/jbc.M110.169953 PMC3075721

[bib31] McClellan A. J. Scott M. D. Frydman J., 2005 Folding and quality control of the VHL tumor suppressor proceed through distinct chaperone pathways. Cell 121: 739–748 15935760 10.1016/j.cell.2005.03.024

[bib32] Meacham G. C. Patterson C. Zhang W. Younger J. M. Cyr D. M., 2001 The Hsc70 co-chaperone CHIP targets immature CFTR for proteasomal degradation. Nat. Cell Biol. 3: 100–105 11146634 10.1038/35050509

[bib33] Meng Z. Lou Z. Liu Z. Li M. Zhao X., 2006 Crystal structure of human pyrroline-5-carboxylate reductase. J. Mol. Biol. 359: 1364–1377 16730026 10.1016/j.jmb.2006.04.053

[bib34] Metzger M. B. Maurer M. J. Dancy B. M. Michaelis S., 2008 Degradation of a cytosolic protein requires endoplasmic reticulum-associated degradation machinery. J. Biol. Chem. 283: 32302–32316 18812321 10.1074/jbc.M806424200 PMC2583311

[bib35] Nillegoda N. B. Theodoraki M. A. Mandal A. K. Mayo K. J. Ren H. Y., 2010 Ubr1 and ubr2 function in a quality control pathway for degradation of unfolded cytosolic proteins. Mol. Biol. Cell 21: 2102–2116 20462952 10.1091/mbc.E10-02-0098 PMC2893976

[bib36] Pickart C. M. Eddins M. J., 2004 Ubiquitin: Structures, functions, mechanisms. Biochim. Biophys. Acta 1695: 55–72 15571809 10.1016/j.bbamcr.2004.09.019

[bib37] Prasad R. Kawaguchi S. Ng D. T., 2010 A nucleus-based quality control mechanism for cytosolic proteins. Mol. Biol. Cell 21: 2117–2127 20462951 10.1091/mbc.E10-02-0111 PMC2893977

[bib38] Qian S. B. McDonough H. Boellmann F. Cyr D. M. Patterson C., 2006 CHIP-mediated stress recovery by sequential ubiquitination of substrates and Hsp70. Nature 440: 551–555 16554822 10.1038/nature04600 PMC4112096

[bib39] Ravid T. Kreft S. G. Hochstrasser M., 2006 Membrane and soluble substrates of the Doa10 ubiquitin ligase are degraded by distinct pathways. EMBO J. 25: 533–543 16437165 10.1038/sj.emboj.7600946 PMC1383530

[bib40] Rosenbaum J. C. Fredrickson E. K. Oeser M. L. Garrett-Engele C. M. Locke M. N., 2011 Disorder targets misorder in nuclear quality control degradation: A disordered ubiquitin ligase directly recognizes its misfolded substrates. Mol. Cell 41: 93–106 21211726 10.1016/j.molcel.2010.12.004 PMC3042722

[bib41] Roth J. Yam G. H. Fan J. Hirano K. Gaplovska-Kysela K., 2008 Protein quality control: The who’s who, the where’s and therapeutic escapes. Histochem. Cell Biol. 129: 163–177 18075753 10.1007/s00418-007-0366-7 PMC2228381

[bib42] Rothstein R., 1991 Targeting, disruption, replacement, and allele rescue: Integrative DNA transformation in yeast. Methods Enzymol. 194: 281–301 2005793 10.1016/0076-6879(91)94022-5

[bib43] Sali A. Blundell T. L., 1993 Comparative protein modelling by satisfaction of spatial restraints. J. Mol. Biol. 234: 779–815 8254673 10.1006/jmbi.1993.1626

[bib44] Shen M. Y. Sali A., 2006 Statistical potential for assessment and prediction of protein structures. Protein Sci. 15: 2507–2524 17075131 10.1110/ps.062416606 PMC2242414

[bib45] Shimma Y. Nishikawa A. bin Kassim B. Eto A. Jigami Y., 1997 A defect in GTP synthesis affects mannose outer chain elongation in *Saccharomyces cerevisiae*. Mol. Gen. Genet. 256: 469–480 9413430 10.1007/s004380050591

[bib46] Sultana R. Theodoraki M. A. Caplan A. J., 2012 UBR1 promotes protein kinase quality control and sensitizes cells to Hsp90 inhibition. Exp. Cell Res. 318: 53–60 21983172 10.1016/j.yexcr.2011.09.010 PMC3221935

[bib47] Tong A. H. Boone C., 2006 Synthetic genetic array analysis in *Saccharomyces cerevisiae*. Methods Mol. Biol. 313: 171–192 16118434 10.1385/1-59259-958-3:171

[bib48] Tongaonkar P. Beck K. Shinde U. P. Madura K., 1999 Characterization of a temperature-sensitive mutant of a ubiquitin-conjugating enzyme and its use as a heat-inducible degradation signal. Anal. Biochem. 272: 263–269 10415098 10.1006/abio.1999.4190

[bib49] Wolf D. H. Schafer A., 2005 CPY^*^ and the power of yeast genetics in the elucidation of quality control and associated protein degradation of the endoplasmic reticulum. Curr. Top. Microbiol. Immunol. 300: 41–56 16573236 10.1007/3-540-28007-3_3

