# Supplementary material for: The Yeast Ubr1 Ubiquitin Ligase Participates in a Prominent Pathway That Targets Cytosolic Thermosensitive Mutants for Degradation
Source: G3 (Bethesda). 2012 May 1;2(5):619–28. doi: 10.1534/g3.111.001933 (PMC3362944; doi:10.1534/g3.111.001933)
Supplement: Supporting Information [file supp_2_5_619__index.html]

Supporting Information 

# The Yeast Ubr1 Ubiquitin Ligase Participates in a Prominent Pathway That Targets Cytosolic Thermosensitive Mutants for Degradation

## Supporting Information for Khosrow-Khavar *et al*, 2012

**Files in this Data Supplement:**

- Table S1 - List of Yeast Strains Used in this Study (PDF, 64 KB)
