# Supplementary material for: The Yeast Ubr1 Ubiquitin Ligase Participates in a Prominent Pathway That Targets Cytosolic Thermosensitive Mutants for Degradation
Source: G3 (Bethesda). 2012 May 1;2(5):619–28. doi: 10.1534/g3.111.001933 (PMC3362944; doi:10.1534/g3.111.001933)
Supplement: Supporting Information [file supp_2.5.619_TableS1.pdf]

Table S1 List of Yeast Strains Used in this Study

Name	Genotype
YTM711	*MAT**a** ura3Δ0 leu2Δ0 his3Δ1 LYS2 MET15 can1Δ::LEU2-MFA1pr::HIS3 grs1-5 -13MYC::KanMX6::URA3*
YTM712	*MAT**a** ura3Δ0 leu2Δ0 his3Δ1 LYS2 MET15 can1Δ::LEU2-MFA1pr::HIS3 grs1-2-13MYC::KanMX6::URA3*
YTM713	*MAT**a** ura3Δ0 leu2Δ0 his3Δ1 LYS2 MET15 can1Δ::LEU2-MFA1pr::HIS3 grs1-3-13MYC::KanMX6::URA3*
YTM714	*MAT**a** ura3Δ0 leu2Δ0 his3Δ1 LYS2 met15Δ0 can1Δ::LEU2-MFA1pr::HIS3 grs1-4-13MYC::KanMX6*
YTM715	*MAT**a** ura3Δ0 leu2Δ0 his3Δ1 LYS2 MET15 can1Δ::LEU2-MFA1pr::HIS3 pro3-1-13MYC::KanMX6::URA3*
YTM716	*MAT**a** ura3Δ0 leu2Δ0 his3Δ1 lys2Δ0 MET15 can1Δ::LEU2-MFA1pr::HIS3 pro3-2-13MYC::KanMX6*
YTM717	*MAT**a** ura3Δ0 leu2Δ0 his3Δ1 lys2Δ0 met15Δ0 can1Δ::LEU2-MFA1pr::HIS3 ugp1-1-13MYC::KanMX6*
YTM718	*MAT**a** ura3Δ0 leu2Δ0 his3Δ1 lys2Δ0 MET15 can1Δ::LEU2-MFA1pr::HIS3 ugp1-2-13MYC::KanMX6::URA3*
YTM719	*MAT**a** ura3Δ0 leu2Δ0 his3Δ1 LYS2 MET15 can1Δ::LEU2-MFA1pr::HIS3 ugp1-3-13MYC::KanMX6::URA3*
YTM720	*MAT**a** ura3Δ0 leu2Δ0 his3Δ1 LYS2 met15Δ0 can1Δ::LEU2-MFA1pr::HIS3 ugp1-4-13MYC::KanMX6::URA3*
YTM721	*MAT**a** ura3Δ0 leu2Δ0 his3Δ1 LYS2 MET15 can1Δ::LEU2-MFA1pr::HIS3 ugp1-5-13MYC::KanMX6::URA3*
YTM722	*MAT**a** ura3Δ0 leu2Δ0 his3Δ1 LYS2 met15Δ0 can1Δ::LEU2-MFA1pr::HIS3 guk1-7-13MYC::KanMX6::URA3*
YTM723	*MAT**a** ura3Δ0 leu2Δ0 his3Δ1 LYS2 met15Δ0 can1Δ::LEU2-MFA1pr::HIS3 guk1-8-13MYC::KanMX6::URA3*
YTM724	*MAT**a** ura3Δ0 leu2Δ0 his3Δ1 lys2Δ0 met15Δ0 can1Δ::LEU2-MFA1pr::HIS3 guk1-9-13MYC::KanMX6*
YTM725	*MAT**a** ura3Δ0 leu2Δ0 his3Δ1 LYS2 met15Δ0 can1Δ::LEU2-MFA1pr::HIS3 guk1-10-13MYC::KanMX6*
YTM726	*MAT**a** ura3Δ0 leu2Δ0 his3Δ1 LYS2 met15Δ0 can1Δ::LEU2-MFA1pr::HIS3 guk1-5-13MYC::KanMX6::URA3*
YTM727	*MAT**a** ura3Δ0 leu2Δ0 his3Δ1 lys2Δ0 MET15 can1Δ::LEU2-MFA1pr::HIS3 guk1-6-13MYC::KanMX6::URA3*
YTM728	*MAT**a** ura3Δ0 leu2Δ0 his3Δ1 LYS2 MET15 can1Δ::LEU2-MFA1pr::HIS3 gus1-1-13MYC::KanMX6::URA3*
YTM729	*MAT**a** ura3Δ0 leu2Δ0 his3Δ1 LYS2 met15Δ0 can1Δ::LEU2-MFA1pr::HIS3 gus1-2-13MYC::KanMX6::URA3*
YTM730	*MAT**a** ura3Δ0 leu2Δ0 his3Δ1 LYS2 met15Δ0 can1Δ::LEU2-MFA1pr::HIS3 gus1-3-13MYC::KanMX6*
YTM731	*MAT**a** ura3Δ0 leu2Δ0 his3Δ1 lys2Δ0 met15Δ0 can1Δ::LEU2-MFA1pr::HIS3 gln1-1-13MYC::KanMX6*
YTM732	*MAT**a** ura3Δ0 leu2Δ0 his3Δ1 lys2Δ0 met15Δ0 can1Δ::LEU2-MFA1pr::HIS3 gln1-2-13MYC::KanMX6*
YTM734	*MAT**a** ura3Δ0 leu2Δ0 his3Δ1 lys2Δ MET15 CAN1 guk1-6-13MYC::KanMX6::URA3*
YTM735	*MAT**a** ura3Δ0 leu2Δ0 his3Δ1 lys2Δ0 met15Δ0 CAN1 gln1-1-13MYC::KanMX6*
YTM736	*MAT**a** ura3Δ0 leu2Δ0 his3Δ1 LYS2 met15Δ0 CAN1 guk1-7-13MYC::KanMX6::URA3*
YTM737	*MAT**a** ura3Δ0 leu2Δ0 his3Δ1 LYS2 MET15 CAN1 gus1-3-13MYC::KanMX6*
YTM738	*MAT**a** ura3Δ0 leu2Δ0 his3Δ1 lys2Δ0 MET15 CAN1 gln1-2-13MYC::KanMX6*
YTM739	*MAT**a** ura3Δ0 leu2Δ0 his3Δ1 LYS2 MET15 CAN1 pro3-1-13MYC::KanMX6::URA3*
YTM741	*MAT**a** ura3Δ0 leu2Δ0 his3Δ1 lys2Δ0 MET15 CAN1 ugp1-3-13MYC::KanMX6::URA3*
YTM742	*MAT**a** ura3Δ0 leu2Δ0 his3Δ1 lys2Δ0 MET15 CAN1 guk1-9-13MYC::KanMX6*
YTM743	*MAT**a** ura3Δ0 leu2Δ0 his3Δ1 lys2Δ0 MET15 CAN1 guk1-8-13MYC::KanMX6::URA3*
YTM744	*MAT**a** ura3Δ0 leu2Δ0 his3Δ1 lys2Δ0 MET15 CAN1 grs1-4-13MYC::KanMX6*
YTM769	*MAT**a** ura3Δ0 his3Δ1, leu2Δ0 LYS2 met15Δ0 CAN1 pro3-1-13MYC::KanMX6::URA3 san1Δ::NatMX6*
YTM770	*MAT**a** ura3Δ0 his3Δ1 leu2Δ0 LYS2 MET15 CAN1 pro3-1-13MYC::KanMX6::URA3 ubr1Δ::NatMX6*
YTM771	*MAT**a** ura3Δ0 his3Δ1 leu2Δ0 LYS2 met15Δ CAN1 pro3-1-13MYC::KanMX6 ubr2Δ::NatMX6*
YTM772	*MATalpha ura3Δ0 his3Δ1 leu2Δ0 LYS2 MET15 CAN1 pro3-1-13MYC::KanMX6 hrd1Δ::NatMX6*

YTM773	*MATalpha ura3Δ0 his3Δ1 leu2Δ0 LYS2 met15Δ CAN1 pro3-1-13MYC::KanMX6::URA3 ubr1Δ::NatMX6 san1Δ::HisMX6*
YTM774	*MAT**a** can1Δ::LEU2-MFA1pr::HIS3* ura3*Δ0 his3Δ1 leu2Δ0 LYS2 MET15 PRO3-13MYC::KanMX6::URA3?*
YTM775	*MAT**a** can1Δ::LEU2-MFA1pr::HIS3* ura3*Δ0 his3Δ1 leu2Δ0 LYS2 MET15 GLN1-13MYC::KanMX6*
YTM776	*MAT**a** can1Δ::LEU2-MFA1pr::HIS3* ura3*Δ0 his3Δ1 leu2Δ0 LYS2 MET15 GUS1-13MYC::KanMX6*
YTM777	*MAT**a** can1Δ::LEU2-MFA1pr::HIS3* ura3*Δ0 his3Δ1 leu2Δ0 LYS2 MET15 GRS1-13MYC::KanMX6*
YTM778	*MAT**a** can1Δ::LEU2-MFA1pr::HIS3* ura3*Δ0 his3Δ1 leu2Δ0 LYS2 MET15 UGP1-13MYC::KanMX6*
YTM779	*MAT**a** can1Δ::LEU2-MFA1pr::HIS3* ura3*Δ0 his3Δ1 leu2Δ0 LYS2 MET15 GUK1-13MYC::KanMX6*
YTM785	*MAT**a*** ura3*Δ0 his3Δ1 leu2Δ0 lys2Δ0 met15Δ0 LYP1/lyp1Δ? gln1-2-13myc::KanMX6 ubr1ΔNatMX6*
YTM786	*MAT**a*** ura3*Δ0 his3Δ1 leu2Δ0 LYS2 MET15 LYP1/lyp1Δ? ugp1-3-13myc::KanMX6::URA3 ubr1ΔNatMX6*
YTM790	*MAT**a*** ura3*Δ0 his3Δ1 leu2Δ0 LYS2 met15Δ0 LYP1/lyp1Δ? pro3-1-13myc::KanMX6::URA3 rkr1ΔNatMX6*
YTM791	*MAT**a*** ura3*Δ0 his3Δ1 leu2Δ0 LYS2 MET15 LYP1/lyp1Δ? can1Δ::STE2pr-spHIS5 pro3-1-13myc::KanMX6::URA3 hul5ΔNatMX6*
YTM794	*MAT**a** his3Δ1 leu2Δ0 LYS2 MET15 pro3-1-13MYC::KanMX6::URA3 CAN1, doa10Δ::HIS3*
YTM1073	*MAT**a** ura3Δ0 leu2Δ0 his3Δ1 lys2Δ0 MET15 CAN1 gln1-2-13MYC::KanMX6 sse1Δ::KanMX6*
